# Understanding the Photoluminescence Quenching of Liquid Exfoliated WS_2_ Monolayers

**DOI:** 10.1021/acs.jpcc.2c05284

**Published:** 2022-12-14

**Authors:** Zhaojun Li, Farnia Rashvand, Hope Bretscher, Beata M. Szydłowska, James Xiao, Claudia Backes, Akshay Rao

**Affiliations:** † Cavendish Laboratory, University of Cambridge, JJ Thomson Avenue, CB3 0HE Cambridge, United Kingdom; ‡ Molecular and Condensed Matter Physics, Department of Physics and Astronomy, Uppsala University, 75120 Uppsala, Sweden; § Institute for Physical Chemistry, Ruprecht-Karls-Universität Heidelberg, Im Neuenheimer Feld 253, 69120 Heidelberg, Germany

## Abstract

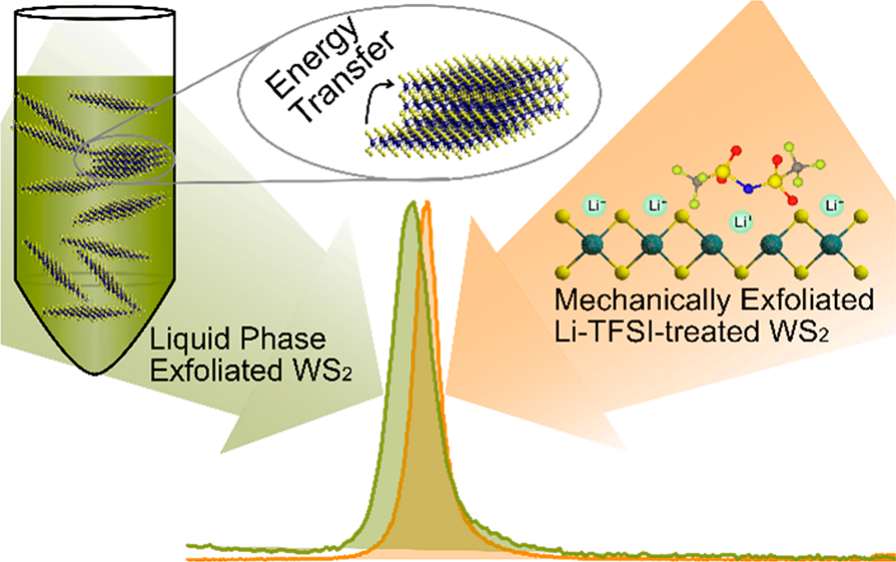

Monolayer transition metal dichalcogenides (TMDs) are being investigated as active materials in optoelectronic devices due to their strong excitonic effects. While mechanical exfoliation (ME) of monolayer TMDs is limited to small areas, these materials can also be exfoliated from their parent layered materials via high-volume liquid phase exfoliation (LPE). However, it is currently considered that LPE-synthesized materials show poor optoelectronic performance compared to ME materials, such as poor photoluminescence quantum efficiencies (PLQEs). Here we evaluate the photophysical properties of monolayer-enriched LPE WS_2_ dispersions via steady-state and time-resolved optical spectroscopy and benchmark these materials against untreated and chemically treated ME WS_2_ monolayers. We show that the LPE materials show features of high-quality semiconducting materials such as very small Stokes shift, smaller photoluminescence line widths, and longer exciton lifetimes than ME WS_2_. We reveal that the energy transfer between the direct-gap monolayers and in-direct gap few-layers in LPE WS_2_ dispersions is a major reason for their quenched PL. Our results suggest that LPE TMDs are not inherently highly defective and could have a high potential for optoelectronic device applications if improved strategies to purify the LPE materials and reduce aggregation could be implemented.

## Introduction

The study of transition metal dichalcogenides (TMDs) has become a vibrant area in nanomaterial science.^1,2^ Exfoliated TMDs have been widely used in the field of optoelectronics due to their excellent light absorptivity and semiconducting performance.^3−6^ To utilize TMD materials, achieving more scalable techniques is critically important, and considerable effort has been devoted to the development of cost-effective mass production methods.^7,8^ Although mechanical exfoliation (ME) produces the highest quality materials, its application is limited by extremely low and uncontrollable yield.^9,10^ In contrast, liquid exfoliation yields atomically thin TMD flakes in a liquid medium in large quantities at moderate cost.^11,12^ The simplest way to produce nanosheets suspended in liquid is termed liquid phase exfoliation (LPE) which relies on immersing the bulk materials into suitable solvents or aqueous surfactant solution and applying high energy, e.g., sonication, to achieve exfoliation accompanied by tearing.^13,14^ When appropriately chosen, the solvent or surfactant suppresses reaggregation in the liquid.^15^ This approach is widely applicable to a range of materials and takes advantage of well-established print production processes for device fabrication.^16,17^

Despite their potential advantages, LPE TMD nanosheets have been rarely used in optoelectronic devices, due to their lower optoelectronic quality in comparison to ME TMDs. A key metric for the optoelectronic quality of a semiconductor is its photoluminescence quantum efficiency (PLQE).^18^ Defects and impurities, as well as chemical and structural inhomogeneity, etc., can lead to nonradiative recombination and hence suppress PLQEs. While it is known that LPE TMDs show poor PLQE, what causes this is unclear. This lack of photophysical understanding is contrasted with great progress in the development of the liquid phase exfoliation methodology and subsequent size selection.^19,20^ Largely defect-free monolayer enriched TMD dispersions with narrow line width photoluminescence (PL), which are similar to that of ME TMDs, have been demonstrated.^21,22^ However, the PLQE of these LPE TMD dispersions remains low and exciton dynamics of TMD dispersions are only little explored.^23^ In particular, most reports focus on ensembles with low monolayer content produced from either LPE or colloidal synthesis.^24−27^ Also, to the best of our knowledge, no reports are making a direct comparison of LPE TMDs vs high-quality ME TMDs, which makes evaluation of the inherent properties of LPE TMDs difficult.

Recently, we showed that chemical treatment of mechanically exfoliated WS_2_ with bis(trifluoromethane)sulfonimide lithium salt (Li-TFSI) allows for greatly suppressed nonradiative decay and trap-free PL emission with intensity over 100 times the untreated monolayers.^28^ In this work, using these high-quality ME systems as a benchmark, we evaluate the quality of monolayer-enriched WS_2_ dispersion produced from LPE. We conduct a systematic study and comparison of the optical and photophysical properties of different LPE WS_2_ dispersions with ME WS_2_ monolayer samples. We find that the LPE WS_2_ dispersions can achieve PL with narrow line width and almost no Stoke shift compared to their absorption spectra. In addition, we use ultrafast pump–probe spectroscopy to study the exciton dynamics following photoexcitation in these WS_2_ samples. We reveal that the energy transfer between monolayers and few-layers in LPE WS_2_ dispersion samples is a major factor in quenching the PL and reducing PLQE yields, suggesting that LPE TMDs can offer very high optoelectronic performance if better size selection can be achieved.

## Methods

### Materials

WS_2_ powder (99%, 2 μm, Sigma-Aldrich), surfactant sodium cholate hydrate (SC, ≥99%), and bis(trifluoromethane)sulfonimide lithium salt (Li-TFSI) are purchased from Sigma-Aldrich and used without purification. The bulk synthetic WS_2_ crystal is purchased from 2D Semiconductors. The mechanically exfoliated monolayer WS_2_ is prepared according to the reported gold-mediated exfoliation method to ensure relatively large monolayers.^29^

### Liquid Exfoliation Process

WS_2_ powder (30 g/L) and SC (8 g/L) are added to a glass bottle with 80 mL of deionized water, and the dispersion is transferred to a stainless-steel beaker for sonication. The beaker is placed in a cooling water bath with a temperature of 5 °C (maintained through a chiller). An ultrasonic replaceable tip is positioned in the dispersion ∼2 cm from the bottom, and the mixture is sonicated for 1 h with 60% amplitude (pulse 8 s on and 2 s off ratio), using a Sonics Vibracell VCX 500, equipped with a threaded probe. The metal beaker is covered with aluminum foil during the sonication process. After the sonication, the dispersion is centrifuged at 6000 rpm for 1.5 h at 8 °C in a Hettich Mikro 220R centrifuge, equipped with a 1016 fixed-angle rotor. The participants are removed afterward and 2 g/L SC solution is added to the dispersion to reach 80 mL, followed by another tip sonication with the same amplitude at 5 °C for 5.5 h. The first sonication step serves the purpose of removing impurities in the WS_2_ powder. After the second sonication, the dispersion is transferred to centrifuge tubes for size selection by liquid cascade centrifugation.^21^ First, the dispersions are centrifuged with relative centrifugal force (RCF) of 5k *g* for 1 h at 8 °C. Supernatant and sediment are separated through manual pipetting. The supernatant is collected and centrifuged at the same speed for 2 h at 8 °C to remove large/thick sheets as completely as possible. Then the supernatant is centrifuged with RCF of 10k *g* for 2 h at 8 °C. The sediment is collected and dispersed in 0.1 g/L SC solution (∼ 2 mL), which is referred to 5–10k *g* WS_2_/H_2_O sample. The supernatant is transferred to new centrifuge tubes for further centrifugation with RCF of 30k *g* for 2 h at 8 °C. In the end, the supernatant is discarded, while the sediment is collected and dispersed in 0.1 g/L SC solution (∼ 2 mL), which is referred to 10–30k *g* WS_2_/H_2_O sample. To transfer the 10–30k *g* WS_2_/H_2_O sample from water to IPA, the dispersion is centrifuged with RCF 30k *g* for 1.5 h to pellet out the nanosheets as sediment and decant the water supernatant. The sediment is redispersed in IPA through 5 min bath sonication. Then the dispersion is centrifuged with RCF of 2k *g* for 20 min to remove the majority of aggregates. The supernatant is collected as a 10–30k *g* WS_2_/IPA sample.

### Chemical Treatment

The chemical treatment with Li-TFSI (0.02 M in methanol) is carried out in the ambient atmosphere. The chemical treatments are achieved by immersing the samples into concentrated solutions of the investigated chemicals for 40 min and blow-drying with a nitrogen gun afterward.

## Results and Discussion

**Figure 1:**
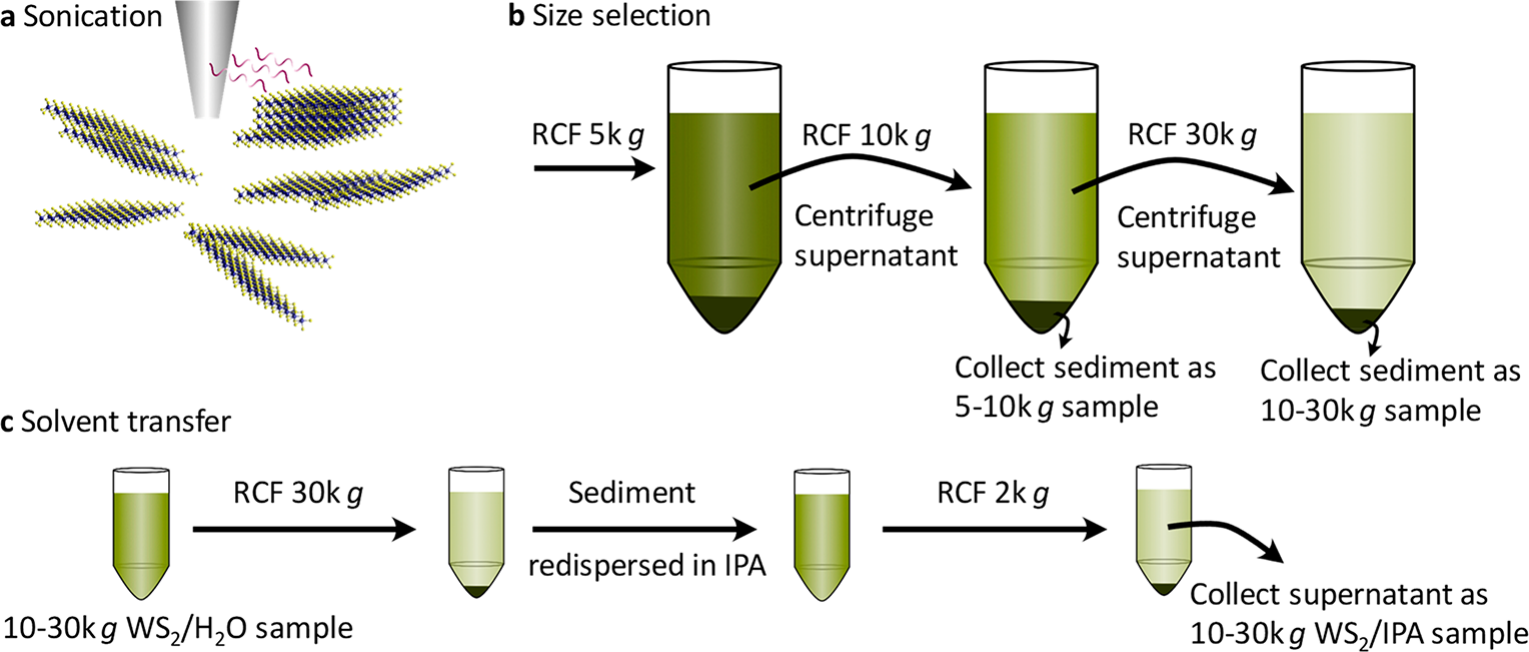
Illustration of liquid exfoliation, size selection, and solvent transfer. (a) Schematic of the tip sonication. (b) Schematic of the liquid cascade centrifugation. Relative centrifugal forces of (RCFs) 5k *g*, 10k *g*, and 30k *g* are used. The supernatant after each step is transferred to another centrifugation at higher centrifugal acceleration, while the sediments are collected. (c) Schematic of the dispersion solvent exchange, transferring from H_2_O to IPA. 30k *g* RCF is used to spin-down the WS_2_ nanosheets as a pellet allowing for solvent exchange. 2k *g* RCF is used to remove aggregated nanosheets as sediments.

The LPE WS_2_ dispersion samples used in this study were prepared as shown in Figure 1. The liquid-suspended WS_2_ nanosheets are generated with the aid of dip sonication and stabilized against reaggregation by the surfactant sodium cholate in water. A size selection process is followed since the as-produced dispersion is highly polydisperse displaying a low monolayer content. Size selection is achieved by liquid cascade centrifugation (LCC) with subsequently increasing rotational speeds.^30^ Heavier and multilayer nanosheets are removed in each step of the LCC process, resulting in more and more monolayer-enriched supernatants. Two size-selected nanosheet distributions are collected as sediments after 10k *g* and 30k *g* centrifugation, hereafter labeled as 5–10k *g* WS_2_/H_2_O sample and 10–30k *g* WS_2_/H_2_O sample, respectively. Isopropyl alcohol (IPA) is also known to give stable dispersions; however, monolayer enrichment has not yet been demonstrated. Hence, a 10–30k *g* WS_2_/IPA sample is also prepared in comparison by replacing the water/surfactant in the 10–30k *g* WS_2_/H_2_O sample with IPA through a centrifugation procedure. The monolayers obtained in this way are around 50 nm as characterized by transmission electron microscopy (TEM) (Figure S1a), which is similar to what was obtained from previous work.^21^ Since mechanical exfoliation renders high-quality TMD monolayers, ME WS_2_ samples are also prepared as a reference to LPE WS_2_ samples. Large monolayer WS_2_ samples (∼200 μm) prepared on quartz substrates with mechanical exfoliation are identified by optical microscopy (Figure S1b). As shown in Figure S1c, both LPE and ME WS_2_ samples on Si/SiO_2_ substrates are characterized by Raman spectroscopy (excitation wavelength 532 nm), confirming the monolayer with characteristic Raman modes of monolayer WS_2_ (e.g., the 2LA(M) at 354 cm^–1^).^31,32^

**Figure 2:**
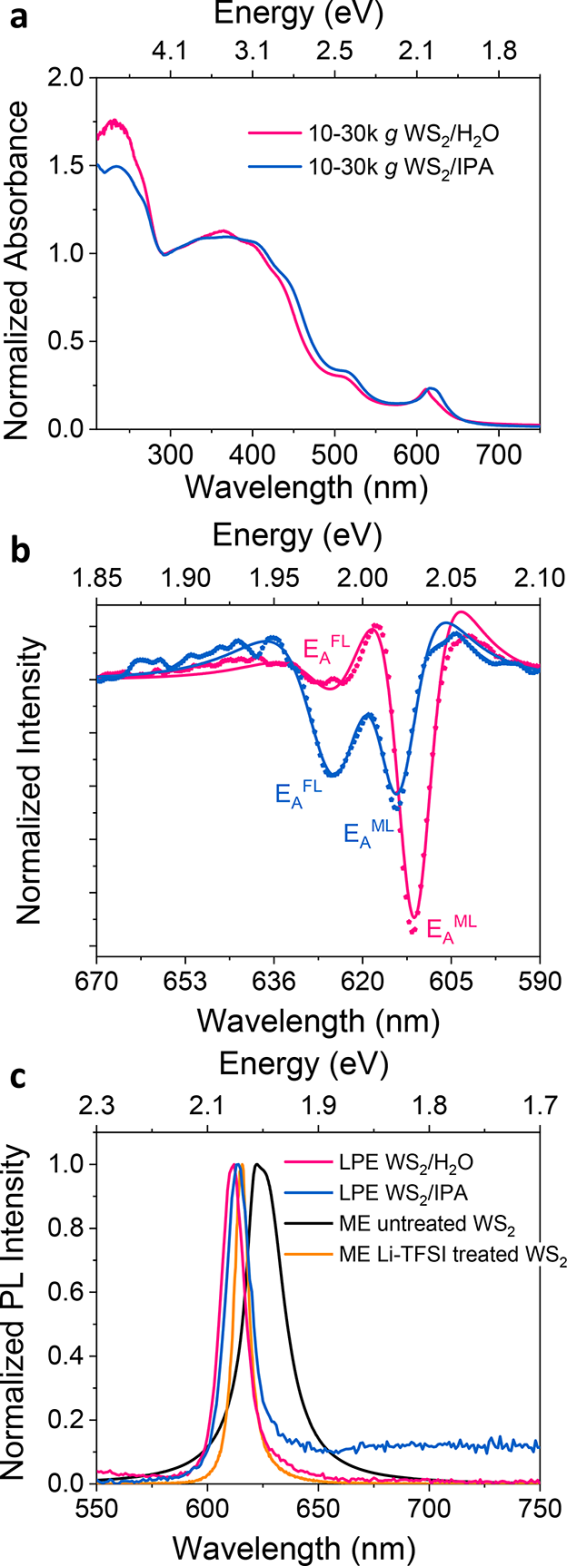
(a) Extinction spectra of the 10–30k *g* WS_2_/H_2_O and 10–30k *g* WS_2_/IPA dispersion samples (normalized to 290 nm). (b) Second derivatives of the A-exciton were obtained after smoothing the spectrum with the Lowess method. The spectra are fitted to the second derivative of two Lorentzians (described in the Supporting Information). The positions of monolayer A-exciton (E_A_^ML^) and few-layer A-exciton (E_A_^FL^) are marked in the figure. (c) Normalized PL spectra of the liquid-exfoliated 10–30k *g* WS_2_/H_2_O and 10–30k *g* WS_2_/IPA dispersion samples as well as mechanically exfoliated untreated and Li-TFSI treated WS_2_ samples.

The optical absorption properties of LPE 5–10k *g* WS_2_/H_2_O, 10–30k *g* WS_2_/H_2_O, and 10–30k *g* WS_2_/IPA dispersions are characterized via UV–vis extinction spectroscopy. The optical properties are summarized in Table 1. The spectra depicted in Figure 2a and Figure S2a are normalized to the local minimum at 290 nm since the extinction coefficient at 290 nm is independent of nanosheet thickness and length.^33^ The absorption spectra are dominated by excitonic features. The A-exciton (E_A_^ML^) for all LPE WS_2_ dispersions is analyzed in more detail using the second derivative of the extinction spectra (Figure 2b and Figure S2b). Due to the previously identified exponential blueshift of the A-exciton with decreasing layer number, two components are visible in the second derivative attributed to the A-exciton of the monolayer (E_A_^ML^) and the unresolvable sum of few-layers (E_A_^FL^).^21,22,34^ WS_2_ dispersions in H_2_O show E_A_^ML^ at 2.029 eV (611 nm), while WS_2_ dispersions in IPA present slightly red-shifted E_A_^ML^ at 2.019 eV (614 nm), which may be attributed to solvatochromism and difference of dielectric disorder.^35^ Since the contributions to the A-exciton absorbance of monolayer and few-layer WS_2_ nanosheets are differentiated, the monolayer content is estimated from the second derivative of the A-exciton absorbance peak according to the previously reported method (described in Supporting Information).^21^ There is a clear increasing monolayer volume fraction (*V*_f_) in water dispersions with increasing RCF, which is 17% for the 5–10k *g* WS_2_/H_2_O sample and 78% for the 10–30k *g* WS_2_/H_2_O sample, respectively. On the other hand, the 10–30k *g* WS_2_/IPA sample shows a moderate *V*_f_ at around 35%, suggesting that some aggregation occurred during the solvent transfer. In the following, we focus on the optical and photophysical properties of the monolayer-enriched LPE 10–30k *g* WS_2_/H_2_O dispersion and 10–30k *g* WS_2_/IPA dispersion samples.

**Table 1:** Summary of Monolayer Volume Fraction (*V*_f_), Position of Monolayer A-Exciton Peak (E_A_^ML^), Center and Full Width at Half-Maximum (fwhm) of PL Spectra, and Position and Average Exciton Lifetime ⟨τ⟩ of A-Exciton Ground State Bleach (GSB) from Pump–Probe Measurements

sample	ML *V*_f_ (%)	E_A_^ML^ (abs, eV)	E_A_^ML^ (PL, eV)	fwhm (PL, meV)	E_A_^ML^ (GSB, eV)	⟨τ⟩ (A_M_-exciton GSB, ps)	⟨τ⟩ (A_FL_-exciton GSB, ps)
LPE 10–30k *g* WS_2_/H_2_O	78	2.029	2.029	19	2.029	481	325
LPE 10–30k *g* WS_2_/IPA	35	2.019	2.019	19	2.019	231	759
ME untreated WS_2_			1.981	44	2.006	6	
ME Li-TFSI treated WS_2_			2.013	10	2.013	90	

To evaluate the quality of LPE WS_2_ samples, we start by comparing the steady-state PL profiles of LPE and ME WS_2_ samples. The PL of ME WS_2_ monolayers is measured with a confocal PL setup, while the LPE samples are measured as dispersions. As shown in Figure 2c, the PL position of ME untreated monolayer WS_2_ sample (E_A_^ML^, PL) is around 1.981 eV with a full width at half-maximum (fwhm) value of around 44 meV, indicating the emission mainly stems from a dominating contribution of trions in the sample.^9^ After Li-TFSI treatment, the PL of ME monolayer WS_2_ is greatly enhanced and the peak position blue-shifts accompanied by a more uniform emission profile due to the suppression of trions and defects, as shown in scatter plots of the peak PL counts versus emission peak position acquired from PL spatial maps (Figure S3). In addition, the Li-TFSI-treated WS_2_ sample exhibits a narrower fwhm of around 10 meV. The PL Stokes shift of the ME Li-TFSI treated WS_2_ sample is primarily related to strain.^36^ This is in good agreement with our previous work showing that Li-TFSI treatment can minimize trap and trion states resulting in intrinsic monolayer properties.^10,28^ The PL positions of both the LPE 10–30k *g* WS_2_/H_2_O dispersion and the 10–30k *g* WS_2_/IPA dispersion coincide with that of the monolayer A-exciton absorbance with almost no Stokes shift, suggesting a high optical quality of the samples with near intrinsic properties, Table 1. However, the monolayer enriched LPE WS_2_ dispersions show extremely low PLQE, less than 0.1% as it is too low to determine accurately with our setup. Turning to the fwhm of the PL from the LPE samples, we find that it is around 19 meV for both LPE WS_2_ samples, which is broader than that of the ME Li-TFSI treated WS_2_ samples (10 meV) but narrower than the untreated ME WS_2_ samples (44 meV). This may be ascribed to polydispersity-induced defect-related broadening of the exciton resonances.^37^ These results hint at the puzzling nature of this system, while the lack of Stokes shift and relatively narrow PL line width from the LPE samples would suggest a reasonably high material quality with much fewer defects on the basal planes compared to untreated ME WS_2_ samples, yet the sample shows very poor PLQE. We note that there is a long PL tail below the bandgap of WS_2_ in the LPE 10–30k g WS_2_/IPA dispersion that is attributed to the larger portion of a few layers (i.e., nonmonolayers) caused by aggregation. The aggregated nonmonolayer materials would not be expected to show high PL as they would not be direct-gap. Hence the presence of PL from them would indicate that they play a larger role than expected in these systems.

**Figure 3:**
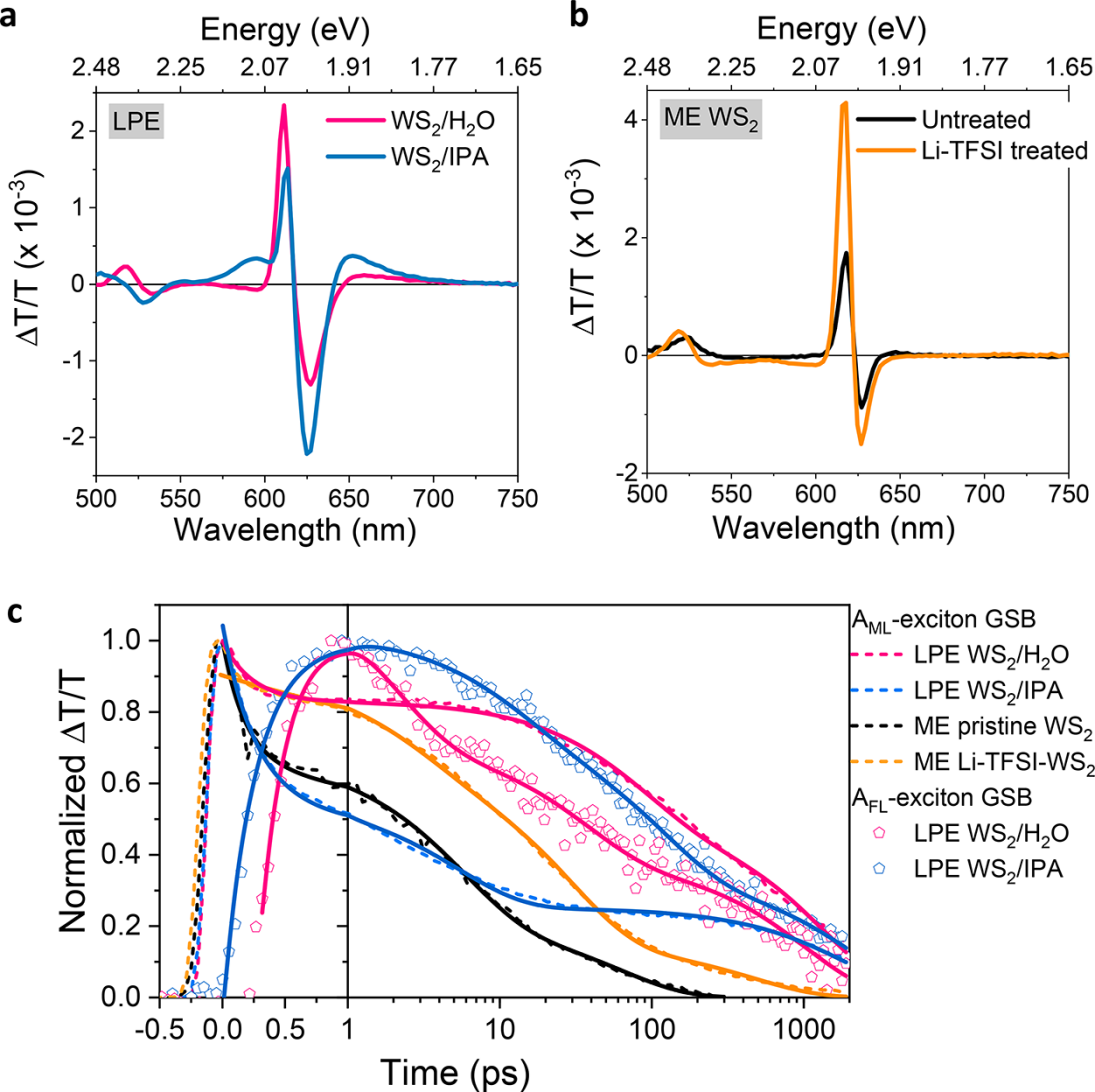
Ultrafast exciton dynamics. Pump–probe spectra at 10 ps time delay (610 nm excitation and 2.63 nJ/pulse) of (a) LPE 10–30k *g* WS_2_/H_2_O and LPE 10–30k *g* WS_2_/IPA samples and (b) ME untreated and Li-TFSI treated WS_2_ monolayer samples. (c) Normalized kinetics taken at the A_ML_-exciton (monolayer) GSB and A_FL_-exciton (few-layer) GSB. Data are well fitted by a three-exponential function (solid lines).

To investigate the reason for the low PLQE of LPE WS_2_ dispersions, we conducted ultrafast pump–probe spectroscopy to explore the exciton dynamics of the LPE WS_2_ dispersions and the ME WS_2_ monolayer samples. Upon excitation, the state filling of the A-exciton leads to a reduction in the ground-state absorption, which is referred to as the A-exciton ground-state bleach (GSB). We record the differential transmission (Δ*T*/*T*) of a white light probe beam as a function of time after photoexcitation by a pulsed laser. The chirp is determined by measuring a blank sample (Figure S4a). The 2D maps of the LPE WS_2_ dispersions and the ME WS_2_ monolayer samples after chirp correction are shown in Figure S4b–e. The full pump–probe spectra of the LPE 10–30k *g* WS_2_/H_2_O dispersion, the WS_2_/IPA dispersion, the ME untreated WS_2_, and the Li-TFSI treated WS_2_ samples excited at around the A-exciton resonance 610 nm (2.033 eV) with 2.63 nJ/pulse are shown in Figure S5. Dynamic screening of Coulomb interaction gives rise to either a comparatively small red-shift or blue-shift of the A-exciton resonance depending on the exciton density.^38,39^ As shown in Figure 3a,b and summarized in Table 1, the monolayer A-exciton GSB maximum (E_A_^ML^, GSB) is located at 611 nm (2.029 eV), 614 nm (2.019 eV), and 616 nm (2.013 eV) for the LPE WS_2_/H_2_O dispersion, the WS_2_/IPA dispersion, and the ME Li-TFSI treated WS_2_ sample, respectively. This coincides with E_A_^ML^ (PL), confirming that the PL of LPE WS_2_ dispersions and ME Li-TFSI treated WS_2_ sample stems from neutral exciton emission, while E_A_^ML^ (GSB) is detected at 618 nm (2.006 eV) for the ME untreated WS_2_ monolayer, which is red-shifted compared to E_A_^ML^ (PL). This is in good agreement with our interpretation that the PL of the ME untreated WS_2_ monolayer is dominated by trion emission. In contrast to the ME monolayers, LPE samples also display a positive feature at around 650–690 nm which we assign to the few-layer A-exciton GSB (E_A_^FL^, GSB). The few-layer signal is more prominent in the LPE WS_2_/IPA sample than that in the 5–10k *g* and 10–30k *g* WS_2_/H_2_O samples (Figures S5 and S6), even though the monolayer content in the WS_2_/IPA dispersion is larger than that in the 5–10k *g* sample. This suggests that this feature is a signature of aggregated nanosheets that is increased in content during the solvent exchange process (detailed discussion in Supporting Information, Table S1, on pages S8–S9).

The normalized kinetics taken at the A_ML_-exciton GSB and A_FL_-exciton GSB are shown in Figure 3c, and the averaged decay lifetimes (⟨τ⟩) are summarized in Table 1 while the fitting results are exhibited in Table S2. The time zero is defined as the maximum intensity of the A_ML_ GSB after chirp correction. It turns out that it is also the time that A_FL_ GSB starts to rise. For the ME untreated monolayer WS_2_ sample, photogenerated excitons decay primarily through nonradiative recombination on a few picoseconds time scale.^40,41^ After Li-TFSI treatment, ⟨τ⟩ increases to tens of picoseconds, which is ascribed to radiative recombination. Surprisingly, the A_ML_-exciton GSB for the LPE 10–30k *g* WS_2_/H_2_O dispersions exhibits longer lifetimes than the high-quality Li-TFSI treated ME samples, again suggesting a high material quality and slower nonradiative recombination.

For both the LPE 10–30k *g* WS_2_/H_2_O and WS_2_/IPA dispersions, A_FL_-exciton GSB rises simultaneously while A_ML_-exciton GSB goes through a fast decay at a time scale of picoseconds. The decay of the A_ML_-exciton GSB and the initial rise and later decay of the A_FL_-exciton GSB are fitted simultaneously with the constraint that the initial decay constant of the A_ML_-exciton GSB and rise of the A_FL_-exciton GSB are the same. Satisfactory fits are obtained with three exponential decays and an additional initial exponential rise for the A_FL_-exciton GSB decays. Importantly, the concomitant rise and decay of mono and multilayer signals indicate that there is energy transfer between monolayers and multilayers in LPE WS_2_ dispersions. This energy transfer from the high-quality direct-gap monolayers to the in-direct gap multilayers may be causing the loss of PL, as the in-direct gap multilayers would be expected to have poor PLQE.

**Figure 4:**
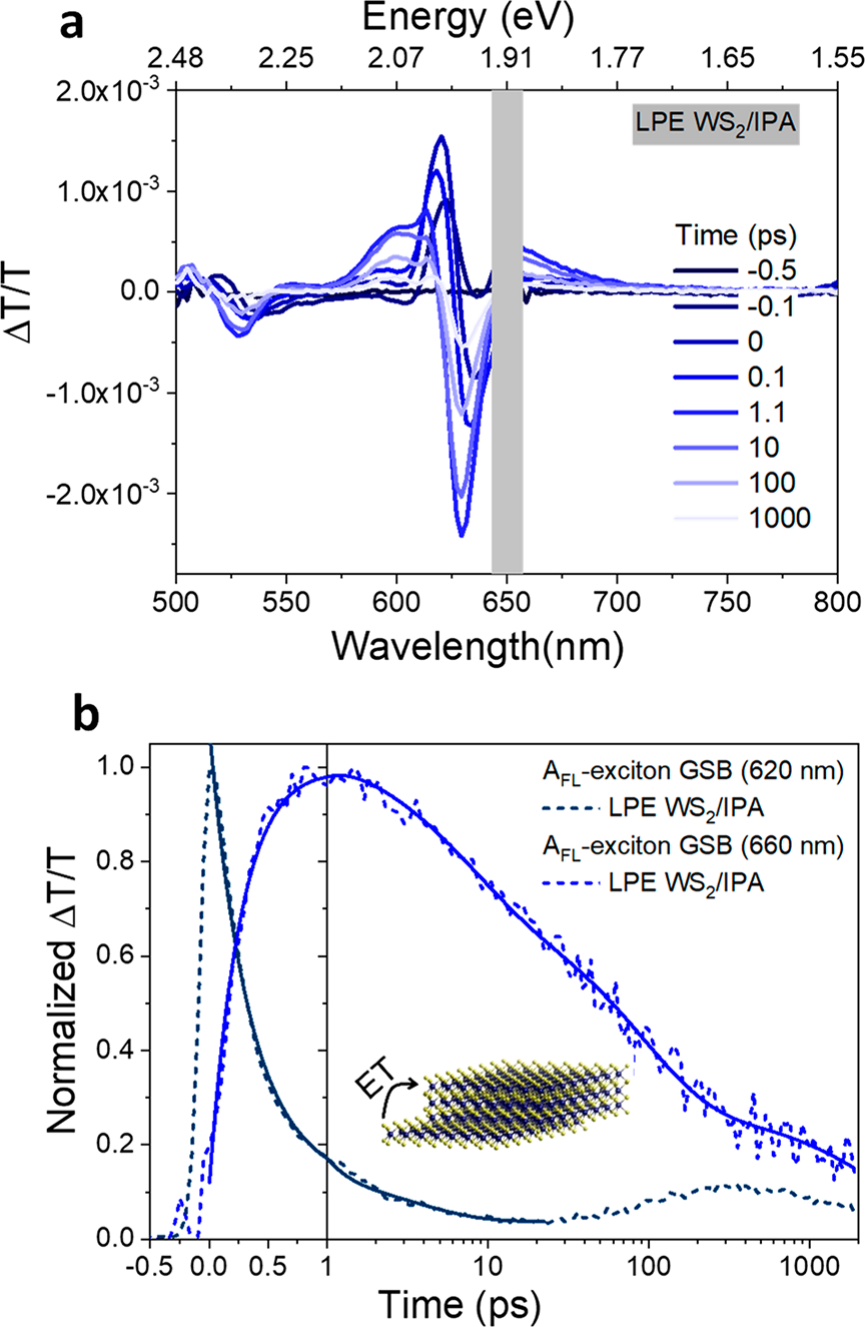
(a) Pump–probe spectra of the LPE 10–30k *g* WS_2_/IPA sample at 650 nm excitation with 5.26 nJ/pulse. (b) Normalized kinetics taken at A_FL_-exciton GSB (620 nm) and A_FL_-exciton GSB (660 nm), illustrating energy transfer (ET) from a few layers to more aggregated multilayers. Data are well fit by three exponential decay functions and an additional rise component for the A_FL_-exciton GSB (660 nm, solid lines). There is a small signal observed before the A_FL_-exciton GSB (660 nm) starts to rise due to the laser scattering.

To test our hypothesis, we conduct further pump–probe measurements by exciting the LPE 10–30k *g* WS_2_/IPA dispersion sample with an energy below the WS_2_ bandgap, at 650 nm, to directly excite the multilayer components and observe the exciton decay. Since the position of A_FL_-exciton GSB red-shifts with increasing layer number, the broad positive features shown in Figure 4a are assigned to the A_FL_-exciton GSB, confirming the existence of lower energy band states due to multilayer nanosheets. The 2D map after chirp correction is shown in Figure S4f. It is worth noting that the positive feature at around 620 nm is actually broader than it appears, due to the bandgap renormalization, and the energy transfer can happen from a few layers to more aggregated multilayers which have a broader A-exciton GSB.^42^ As shown in Figure 4b and summarized in Table S3, simultaneous with the fast A_FL_-exciton (∼620 nm) decay is the growth of more aggregated A_FL_-exciton (∼660 nm) GSB. The further extended ⟨τ⟩ at A_FL_-exciton GSB (∼660 nm) supports our hypothesis that there is energy transfer between the individual sheets in the LPE WS_2_ dispersion. In this scenario, we propose that the optical quality and specifically the PLQE of the LPE WS_2_ can potentially be improved by reducing the energy transfer by introducing a coating on the nanosheets, for example, through chemical functionalization or adsorption of bulky molecules or polymers.

To gain further insight into the exciton dynamics of the LPE WS_2_ samples, we also analyze the photon energy dependence and pump fluence dependence of the A-exciton GSB decay feature (Figures S7–S10; Tables S4–S7). A subpicosecond decay component in the excited-state dynamics of WS_2_ emerges for incident photon energies above the A-exciton resonance. This originates from a nonequilibrium population of charge carriers that form excitons as they cool and is dependent on the photon energy.^42^ Nevertheless, the exciton decays for LPE WS_2_ samples and ME untreated WS_2_ samples are largely independent of the pump fluence (Figures S7 and S8). The fluence-independent nature of the recombination indicates that it is linked to defect-assisted decay,^43^ while the ⟨τ⟩ at A_ML_-exciton GSB for ME Li-TFSI treated WS_2_ samples shortens with the increase of pump fluence due to the enhanced EEA process. This suggests that there are also other reasons for the low PLQE of LPE WS_2_ dispersions besides energy transfer, such as edge effects related to the small lateral dimensions of the monolayers in LPE dispersions. Hence the liquid exfoliation process needs to be further improved to produce samples suitable for practical optoelectronic application.^33^

In conclusion, monolayer-enriched WS_2_ dispersions produced by liquid phase exfoliation (LPE) combined with size selection and WS_2_ monolayers by mechanical exfoliation (ME) were prepared, and the photophysical properties of the samples were compared with steady-state and time-resolved optical spectroscopy. Our results reveal that even though LPE monolayer-enriched WS_2_ dispersions can exhibit pristine-like excitonic features, such as narrow linewidth PL and minimal Stoke shift, the photoluminescence quantum efficiency (PLQE) is very low (<0.1%). Detailed analysis of the exciton dynamics of LPE WS_2_ dispersions through pump–probe spectroscopy suggests that this poor PLQE results from energy transfer between individual monolayer and multilayer nanosheets in the LPE WS_2_ dispersions. The energy transferred to the in-direct gap multilayers then leads to nonradiative recombination. Other factors increasing nonradiative recombination and hence lowering PLQE may include the small lateral sheet size of the LPE samples. Our results suggest that LPE TMDs are not inherently highly defective and could have a high potential for optoelectronic device applications. Instead, aggregation and energy transfer from monolayer to aggregated materials are the cause of the poor PLQE. We, therefore, propose that improved strategies to purify the LPE materials and reduce aggregation could significantly improve the PLQE of these materials. Thicker coatings on the sheets, such as polymers as additional stabilizers, could also potentially reduce the energy transfer phenomenon.

## Data Availability

Data are available in the University of Cambridge data repository https://doi.org/10.17863/CAM.91540.

## Supporting Information Available

Additional experimental details and additional data for optical and photophysical characterization of WS_2_ samples (PDF)
