# Supplementary material for: Understanding the Photoluminescence Quenching of Liquid Exfoliated WS2 Monolayers
Source: J Phys Chem C Nanomater Interfaces. 2022 Dec 14;126(51):21681–8. doi: 10.1021/acs.jpcc.2c05284 (PMC9806825; doi:10.1021/acs.jpcc.2c05284)
Supplement: Supplementary file 1 — jp2c05284_si_001.pdf [file jp2c05284_si_001.pdf]

Supporting Information

Understanding the Photoluminescence Quenching of Liquid Exfoliated WS_2 Monolayers

Zhaojun Li[1,2], Farnia Rashvand[3], Hope Bretscher[1], Beata M. Szydłowska[3], James Xiao[1], Claudia Backes[3,4], Akshay Rao[1*]

[1]Cavendish Laboratory, University of Cambridge, JJ Thomson Avenue, CB3 0HE, Cambridge, United Kingdom

[2]Molecular and Condensed Matter Physics, Department of Physics and Astronomy, Uppsala University, 75120 Uppsala, Sweden

[3]Institute for Physical Chemistry, Ruprecht-Karls-Universität Heidelberg, Im Neuenheimer Feld 253, 69120 Heidelberg, Germany

[4]Current address: Physical Chemistry of Nanomaterials, University of Kassel, Heinrich-Plett-Str.40, D-34132 Kassel, Germany

[*]Corresponding author

Contact e-mail: ar525@cam.ac.uk

Contents

1. Experimental Details 2
2. Calculation of Monolayer Content 3
3. Material characterization 4
4. Optical properties of LPE WS_2 dispersions and ME WS_2 samples 5
5. Additional Pump-probe data on WS_2 samples 6
6. Reference 16

1. Experimental Details

Si/SiO_2 substrates with 90 nm oxide layer were used for microscope steady-state photoluminescence (PL) and Raman spectroscopy. Quartz substrates were used for ultrafast pump-probe measurement. The samples were encapsulated for ultrafast pump-probe measurements, and other measurements are carried out on samples without encapsulation.

Transmission electron microscopy (TEM) was performed using an FEI Tecnai F20 at 200kV accelerating voltage. 10-30k *g* WS_2/H_2O dispersion sample was transferred onto 200-mesh Cu grids (Agar AGS160). The optical microscopy was measured using Olympus BX60 optical microscope with 405 nm laser. Samples were placed on an X-Y piezo stage of the microscope and the signal is collected in reflection mode with the 50× objective. The Raman spectroscopy was carried out on a Renishaw inVia Raman confocal microscope with a 532 nm excitation laser in air under ambient condition. The Raman emission was collected by a 20× long working distance objective lens in streamline mode and dispersed by a 1800 l/mm grating with 1% of the laser power (< 10 μW). The spectrometer was calibrated to a silicon reference sample prior to the measurement to correct for the instrument response.

UV-Vis measurement was performed using a Shimadzu UV-3600 Plus spectrometer to measure the extinction spectra of the LPE WS_2 dispersions in transmission mode in quartz cuvettes with 1 nm increments. Steady-state PL measurement were carried out using a temperature and current-controlled 405 nm laser diode (Thorlabs). The incident beam was attenuated as desired and focused onto the sample while PL from the sample was collected and focused into an Andor Kymera 328i Spectrometer and spectra recorded using a Si-CCd (Andor iDus 420).

The microscope steady-state PL measurement was carried out using a WITec alpha 300 s setup as has been described previously.[1] Importantly, a 405 nm continuous wave laser (Coherent CUBE) was used as the excitation source. A long pass filter with a cutoff wavelength of 450 mm was fitted before signal collection to block excitation scatter. The light was coupled with an optical fiber to the microscope and focused using a 20× Olympus lens. Samples were placed on an X-Y piezo stage of the microscope. The PL signal was collected in refection mode with the same 20× objective and detected using a Princeton Instruments SP-2300i spectrometer fitted with an Andor iDus 401 CCD detector. The PL was measured at 405 nm excitation with a fluence of 15 W cm^{-2}.

The ultrafast pump-probe setup has been described previously.[2] A Light Conversion PHAROS laser system with 400 μJ per pulse at 1030 nm with a repetition rate of 38 kHz is split in two, one part is used to generate the continuum probe light and the second part is used in an Collinear Optical Parametric Amplifier (Orpheus, Light Conversion) to generate the pump source at the desired wavelength. The probe pulse is delayed up to 2 ns with a mechanical delay-stage (Newport). A mechanical chopper (Thorlabs) is used to create an on-off pump-probe pulse series. A silicon line scan camera (JAI SW-2000M-CL-80) fitted onto a visible spectrograph (Andor Solis, Shamrock) is used to record the transmitted probe light.

2. Calculation of Monolayer Content

The monolayer volume fraction (V_f) can be extracted from the extinction spectra of the dispersion according to the previously reported method.[3] The A-exciton extinction is first deconvoluted into components of monolayered and multi-layered WS_2 with differentiation after smoothing the spectra by the Lowess method (10-15 points). The smoothing function suppresses spectral noise well without changing peak shapes. In the simplest form, a Lorentzian line can be described by

$$L(E) = \frac{h}{\left[1 + \left(\frac{(E - E_0)}{w/2}\right)^2\right]} \#(S1)$$

Where h represents the height, E_0 the center and w the full width half maximum (FWHM). Differentiating twice with respect to E gives

$$\frac{d^2L(E)}{dE^2} = -\frac{8h}{w^2}\left[\frac{1 - 3\left(\frac{E - E_0}{w/2}\right)^2}{\left(1 + \left(\frac{E - E_0}{w/2}\right)^2\right)^3}\right] \#(S2)$$

The obtained spectrum of the second derivative is then fitted to the sum of the second derivative of two Lorentzian functions giving E, w and h of the monolayer and few-layer WS_2. The area under the monolayer (ML) A-exciton extinction peak should scale with the monolayer content in the dispersion. As the area under any Lorentzian is proportional to $h \times w$, a metric S_A which scales with the V_f can be calculated as the equation:

$$S_A = \frac{h_{ML}w_{ML}}{h_{ML}w_{ML} + h_{FL}w_{FL}} \#(S3)$$

V_f is then calculated as:[3]

$$V_f = (1.25 \pm 0.08)S_A \#(S4)$$

3. Material characterization

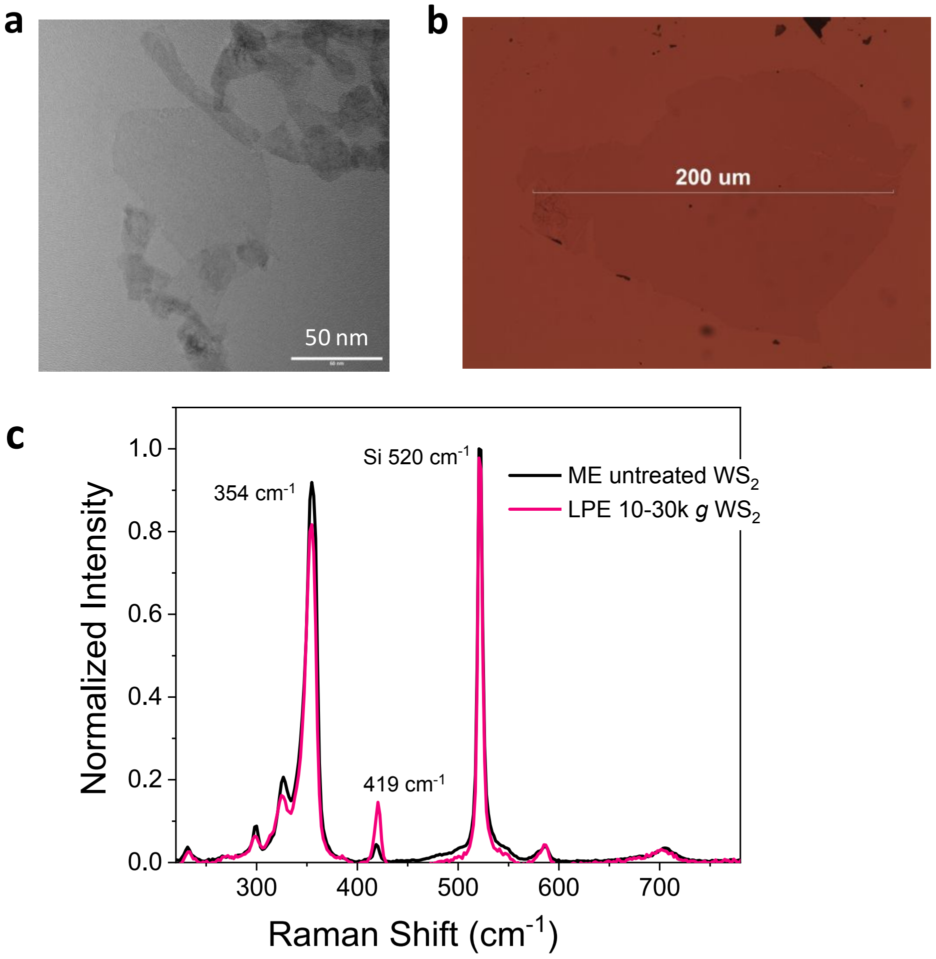

Figure S1. Characterization of liquid exfoliated and mechanically exfoliated WS_2 samples. **a** TEM image of liquid phase exfoliated 10-30k *g* WS_2 sample. **b** Optical microscope image of mechanically exfoliated WS_2 sample on quartz substrate. **c** Raman spectroscopy of liquid phase exfoliated and mechanically exfoliated WS_2 samples on Si/SiO_2 substrate confirming the monolayer.

4. Optical properties of LPE WS_2 dispersions and ME WS_2 samples

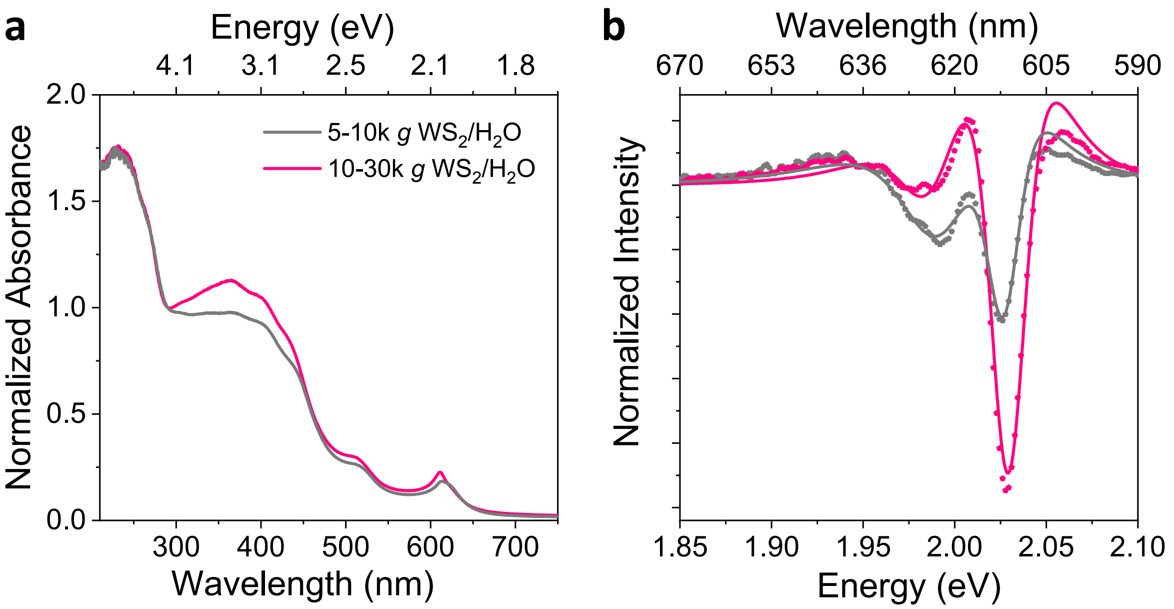

Figure S2. **a** Extinction spectra normalized to 290 nm of the 5-10k *g* and 10-30k *g* WS_2/H_2O samples. **b** Second derivatives of the A-exciton obtained after smoothing the spectrum with the Lowess method. The spectra are fitted to the second derivative of two Lorentzians (solid lines).

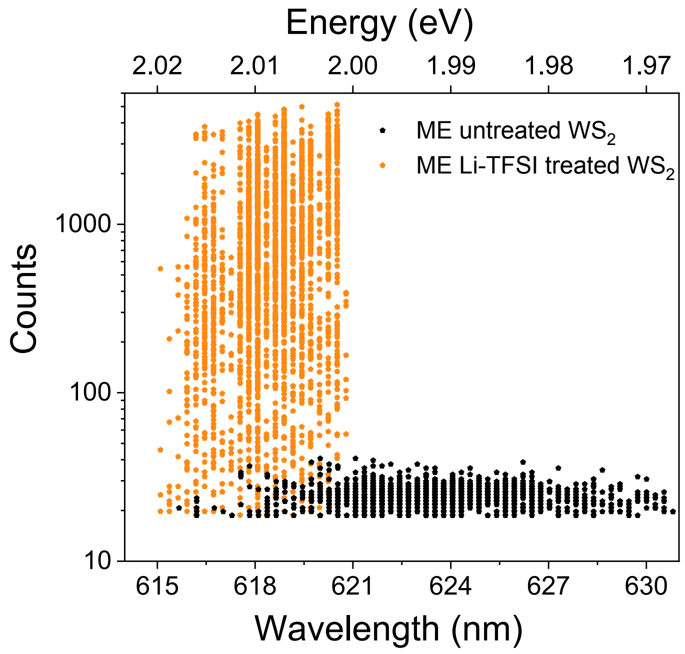

Figure S3. PL scatter plots of spectral position of the peak emission as well as peak untreated and Li-TFSI-treated monolayer WS_2 PL counts extracted from PL maps of WS_2 monolayer on Si-SiO_2 (90 nm) substrate.

5. Additional Pump-probe data on WS_2 samples

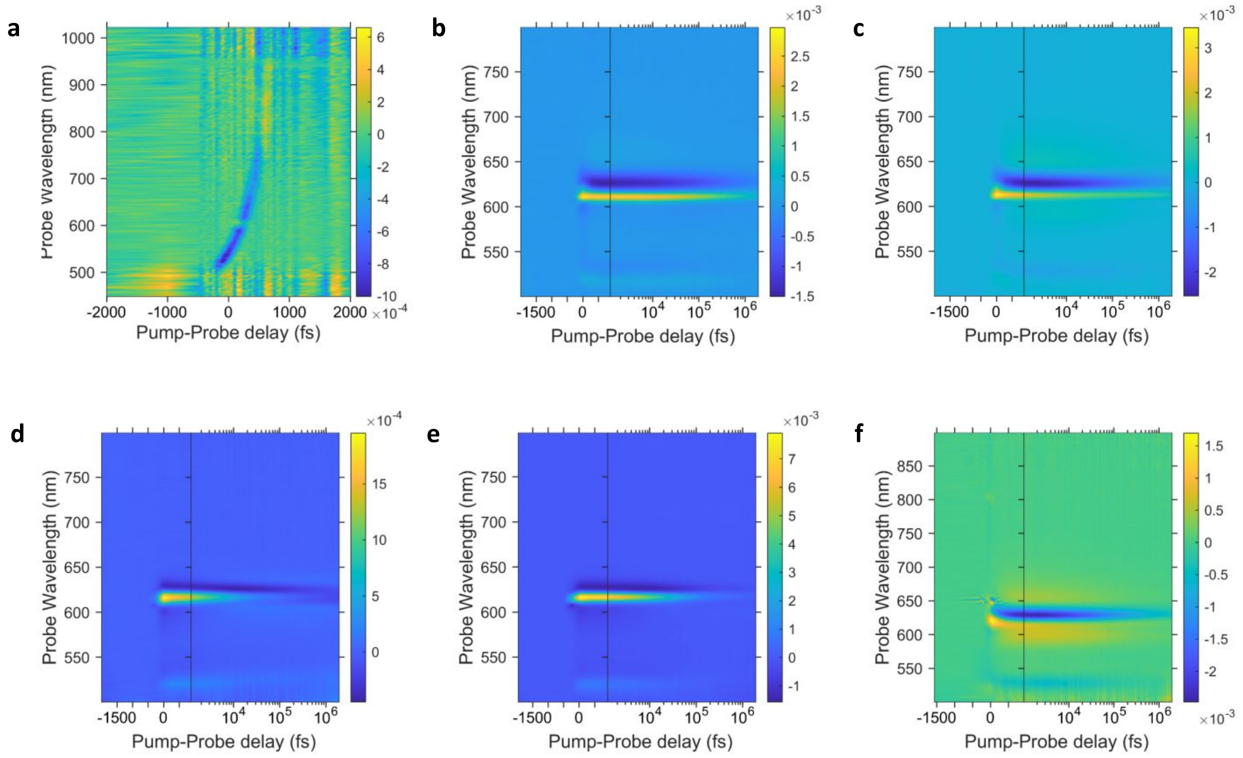

Figure S4. Pump-probe 2D maps. **a** the blank solution sample. **b** liquid phase exfoliated 10-30k g WS_2/H_2O (excited at 610 nm, 2.63 nJ/pulse), **c** liquid phase exfoliated 10-30k g WS_2/IPA (excited at 610 nm, 2.63 nJ/pulse), **d** mechanically exfoliated untreated WS_2 monolayer sample (excited at 610 nm, 2.63 nJ/pulse), and **e** mechanically exfoliated Li-TFSI treated WS_2 monolayer sample (excited at 650 nm, 5.26 nJ/pulse).

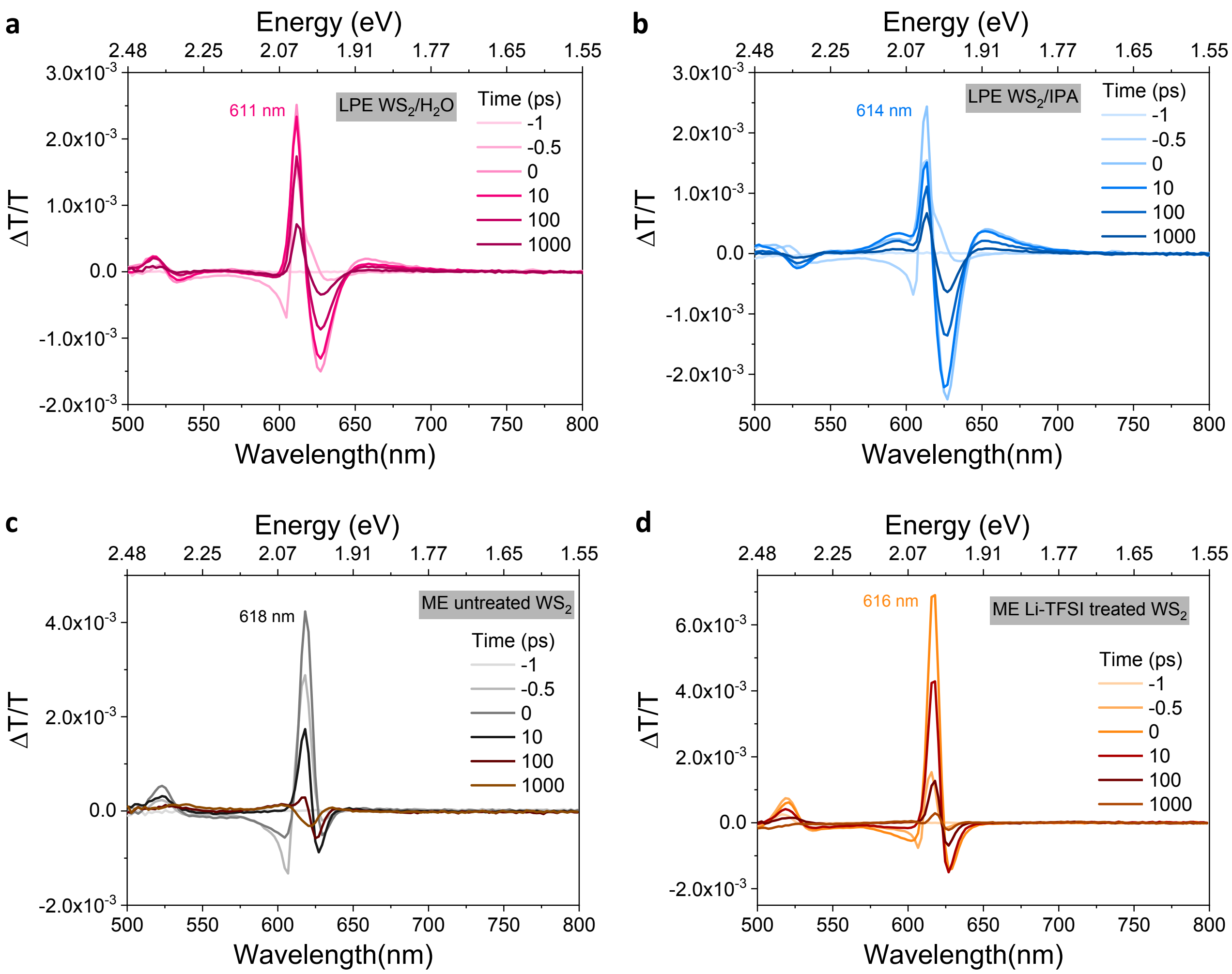

Figure S5. Pump-probe spectra (excited at 610 nm, 2.63 nJ/pulse) of **a** liquid exfoliated 10-30k *g* WS_2/H_2O, **b** liquid phase exfoliated 10-30k *g* WS_2/IPA, **c** mechanically exfoliated untreated WS_2 monolayer sample, and **d** mechanically exfoliated Li-TFSI treated WS_2 monolayer sample.

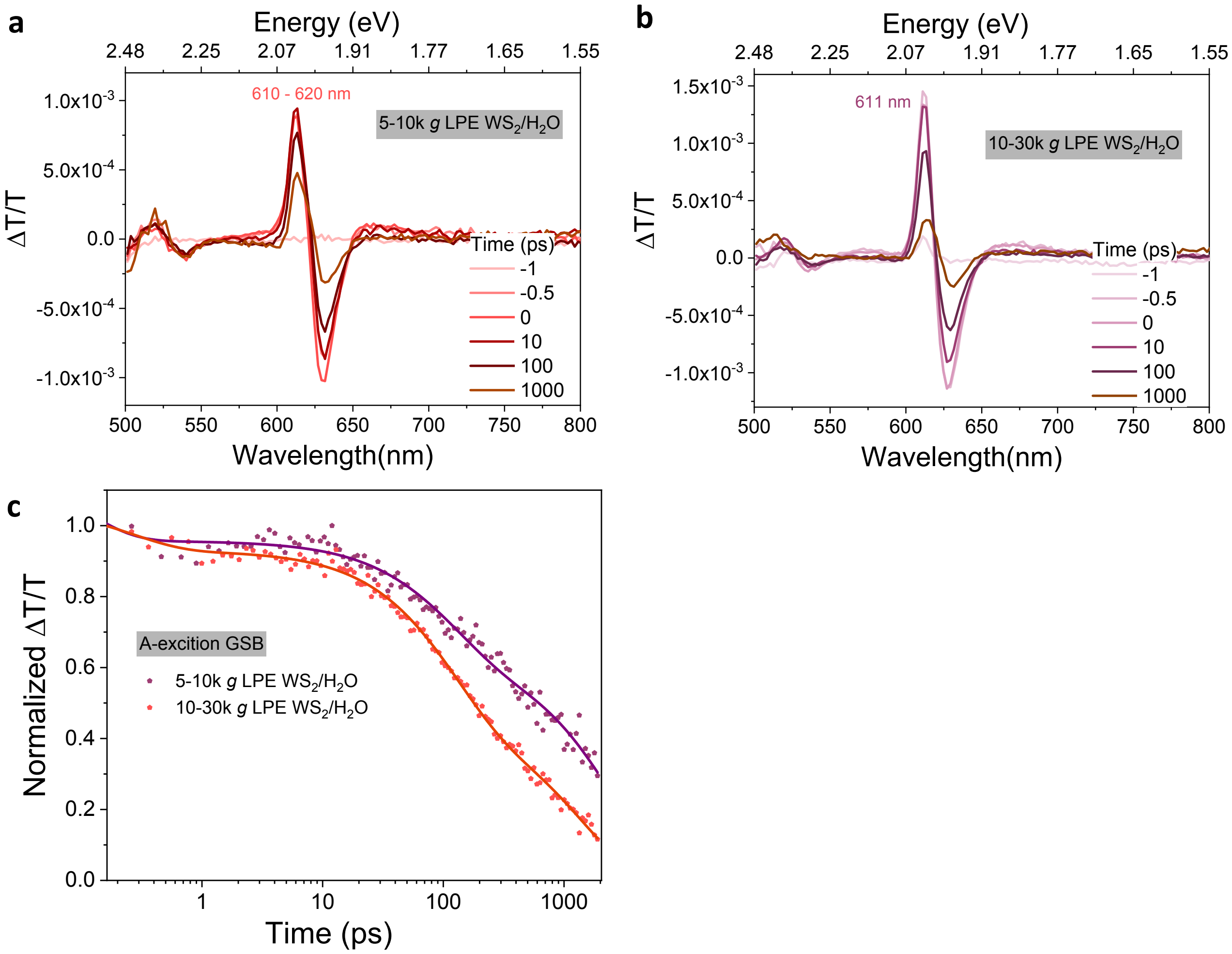

Figure S6. Pump-probe spectra (excited at 500 nm, 0.53 nJ/pulse) of **a** liquid phase exfoliated 5-10k *g* WS_2/H_2O sample, **b** liquid phase exfoliated 10-30k *g* WS_2/ H_2O sample. c Normalized kinetics of A-exciton GSB of both samples.

Table S1. Fitting results of the rates for LPE 10-30k *g* WS_2/IPA sample at 500 nm excitation with 0.53 nJ/pulse in pump-probe measurement.

Sample	A_1	τ_1 (ps)	A_2	τ_2 (ps)	A_3	τ_3 (ps)	$<\tau>$ (ps)
LPE 5-10k *g* WS_2/H_2O A_{ML}-exciton GSB (~611 nm)	0.25	0.10	0.32	114	0.63	2591	1391
LPE 10-30k *g* WS_2/ H_2O A-exciton GSB (610~620 nm)	0.13	0.26	0.48	116	0.45	1419	655

Compared to the LPE 10-30k *g* WS_2/IPA dispersion, both 5-10k *g* and 10-30k *g* WS_2/H_2O show less positive features in the 650 680 nm region in the pump-probe spectra, suggesting the fraction of

multilayers are less than that of WS_2/IPA samples (Figure S4 and S5). This is understandable for 10-30k *g* WS_2/H_2O sample since the V_f is really high, around 78%, however, the V_f in 5-10k *g* WS_2/H_2O sample is quite low (~ 15%). On the other hand, compared to the narrow linewidth of A_{ML}-exciton GSB for both LPE 10-30k *g* WS_2/H_2O and WS_2/IPA, the 5-10k *g* sample shows wider linewidth and even peak splitting in the longer time delay stage. In addition, the A_{ML}-exciton GSB of the 5-10k g sample decays slower than that LPE 10-30k *g* WS_2/H_2O (Figure S5 c and Table S1), indicating there are multilayers which are not distinguishable from monolayer in 5-10k *g* sample and possible energy transfer between them. Therefore, we assume the multilayers existing in 5-10k *g* WS_2/H_2O and 10-30k *g* WS_2/IPA are different. There are more double or triple layers in 5-10k *g* WS_2/H_2O sample which is removed with high centrifugation speed, while there are more aggregated multilayers in 10-30k *g* WS_2/IPA sample due to the reaggregation occurring during the solvent exchange process.

Table S2. Fitting results for the rates at 610 nm excitation with 2.63 nJ/pulse in pump-probe measurement.

Sample	A_1	τ_1 (ps)	A_2	τ_2 (ps)	A_3	τ_3 (ps)	A_4	τ_4 (ps)	$<\tau>$ (ps)
LPE 10-30k *g* WS_2/H_2O A_{ML}-exciton GSB	0.65	0.23	0.34	82	0.49	1396	/	/	481
LPE 10-30k *g* WS_2/H_2O A_{FL}-exciton GSB	-3.6	0.23	0.54	1.8	0.33	35	0.38	1034	360
LPE 10-30k *g* WS_2/IPA A_{ML}-exciton GSB	1.64	0.26	0.32	5.3	0.25	2032	/	/	231
LPE 10-30k *g* WS_2/IPA A_{FL}-exciton GSB	-3.6	0.26	0.26	12	0.43	111	0.34	2151	759
ME untreated WS_2 A_{ML}-exciton GSB	0.74	0.18	0.18	5.5	0.08	63	/	/	6
ME Li-TFSI treated WS_2 A_{ML}-exciton GSB	0.29	2.7	0.55	29	0.16	466	/	/	90

Table S3. Fitting results for the rates for LPE 10-30k *g* WS_2/IPA sample at 650 nm excitation with 5.26 nJ/pulse in pump-probe measurement.

Sample	A_1	τ_1 (ps)	A_2	τ_2 (ps)	A_3	τ_3 (ps)	A_4	τ_4 (ps)	$<\tau>$ (ps)
LPE 10-30k *g* WS_2/IPA A_{FL}-exciton GSB (~620 nm)	2.64	0.28	0.27	1.5	0.04	367	/	/	6
LPE 10-30k *g* WS_2/IPA A_{ML}-exciton GSB (~660 nm)	-2.95	0.28	0.35	6.7	0.45	89	0.27	3156	836

Due to the bandgap renormalization, there is increasing ΔT/T after 10 ps, therefore, the AFL-exciton GSB at 620 nm is fitted to 10 ps. The τ_3 which is determined to be 367 ps is larger than the time for fitting, only indicates that there is a long lived component beyond 10 ps.

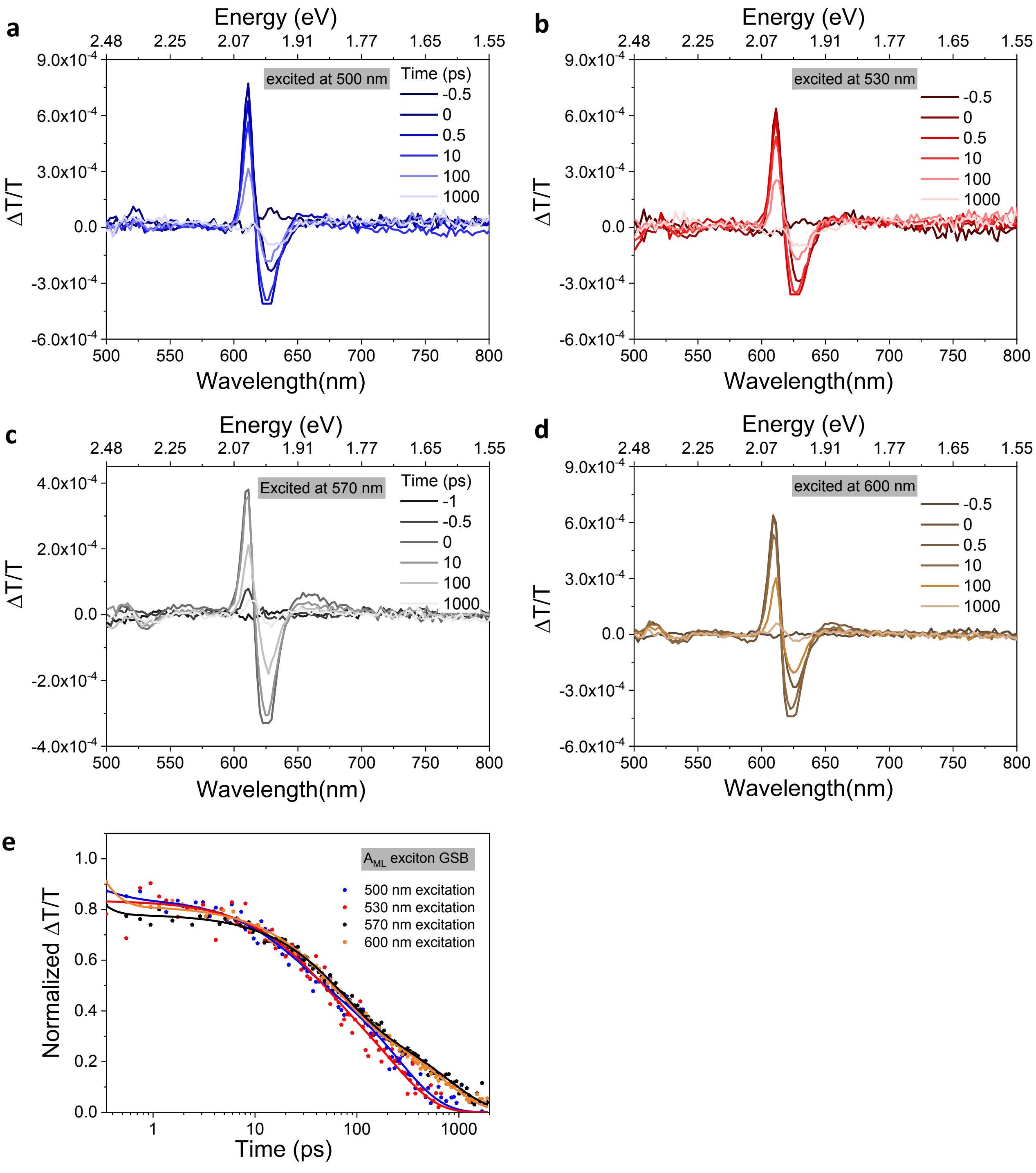

Figure S7. Photon energy dependence of A-exciton resonance with 0.53 nJ/pulse in pump-probe measurements. **a** pump-probe data of the 10-30k *g* WS_2/H_2O sample excited at **a** 500 nm, **b** 530 nm, **c** 570 nm, and **d** 600 nm. **e** Normalized kinetics of A_{ML}-exciton GSB of all samples.

Table S4. Fitting results for the rates of the 10-30k *g* WS_2/H_2O sample with 0.53 nJ/pulse in pump-probe measurement.

Sample	A_1	τ_1 (ps)	A_2	τ_2 (ps)	A_3	τ_3 (ps)	$<\tau>$ (ps)
500 nm excitation	0.16	0.2	0.28	21.7	0.57	254	149
530 nm excitation	0.15	0.07	0.29	30.5	0.54	223	132
570 nm excitation	1.2	0.1	0.42	65.2	0.36	763	153
600 nm excitation	1.4	0.1	0.44	65.1	0.38	655	125

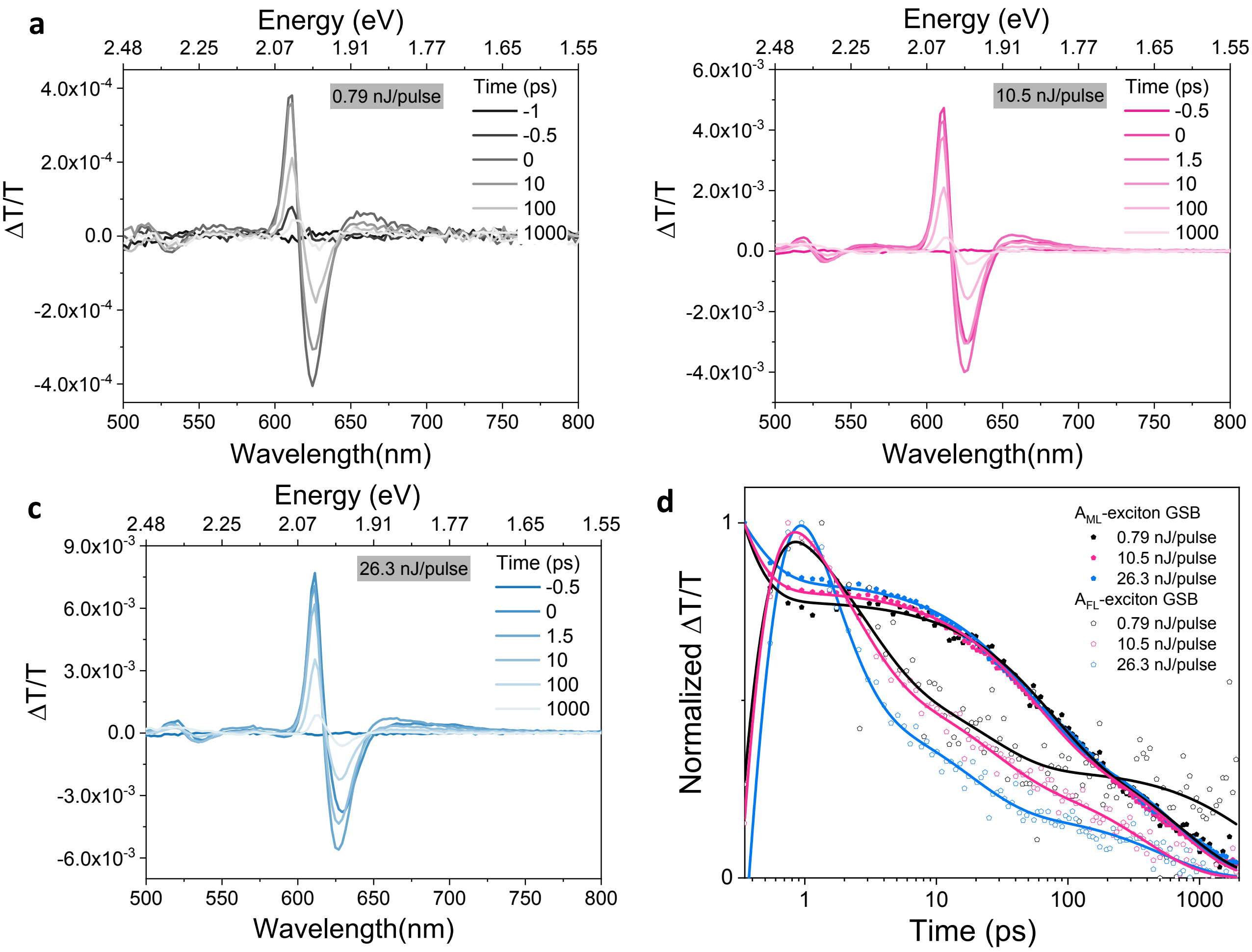

Figure S8. Fluence dependence of A-exciton resonance (excited at 570 nm). **a** pump-probe data of the 10-30k *g* WS_2/H_2O sample excited with **a** 0.79 nJ/pulse, **b** 10.5 nJ/pulse, and **c** 26.3 nJ/pulse. **d** Normalized kinetics of A_{ML}-exciton GSB and A_{FL}-exciton GSB of all samples.

Table S5. Fitting results for the rates at 570 nm excitation of the 10-30k *g* WS_2/H_2O sample in pump-probe measurement.

Sample	A_1	τ_1 (ps)	A_2	τ_2 (ps)	A_3	τ_3 (ps)	A_4	τ_4 (ps)	$\langle\tau\rangle$ (ps)
0.79 nJ/pulse A_{ML}-exciton GSB	2.1	0.14	0.42	66	0.36	766	/	/	106
0.79 nJ/pulse A_{FL}-exciton GSB	-8.9	0.14	0.57	2.6	0.27	24	0.31	2692	733
10.5 nJ/pulse A_{ML}-exciton GSB	1.97	0.15	0.43	51	0.38	667	/	/	99
10.5 nJ/pulse A_{FL}-exciton GSB	-10.2	0.15	0.69	2.0	0.28	24	0.28	386	93
26.3 nJ/pulse A_{ML}-exciton GSB	0.8	0.2	0.44	47	0.4	685	/	/	180
26.3 nJ/pulse A_{FL}-exciton GSB	-8.29	0.2	1.32	1.3	0.3	17.6	0.19	518	58

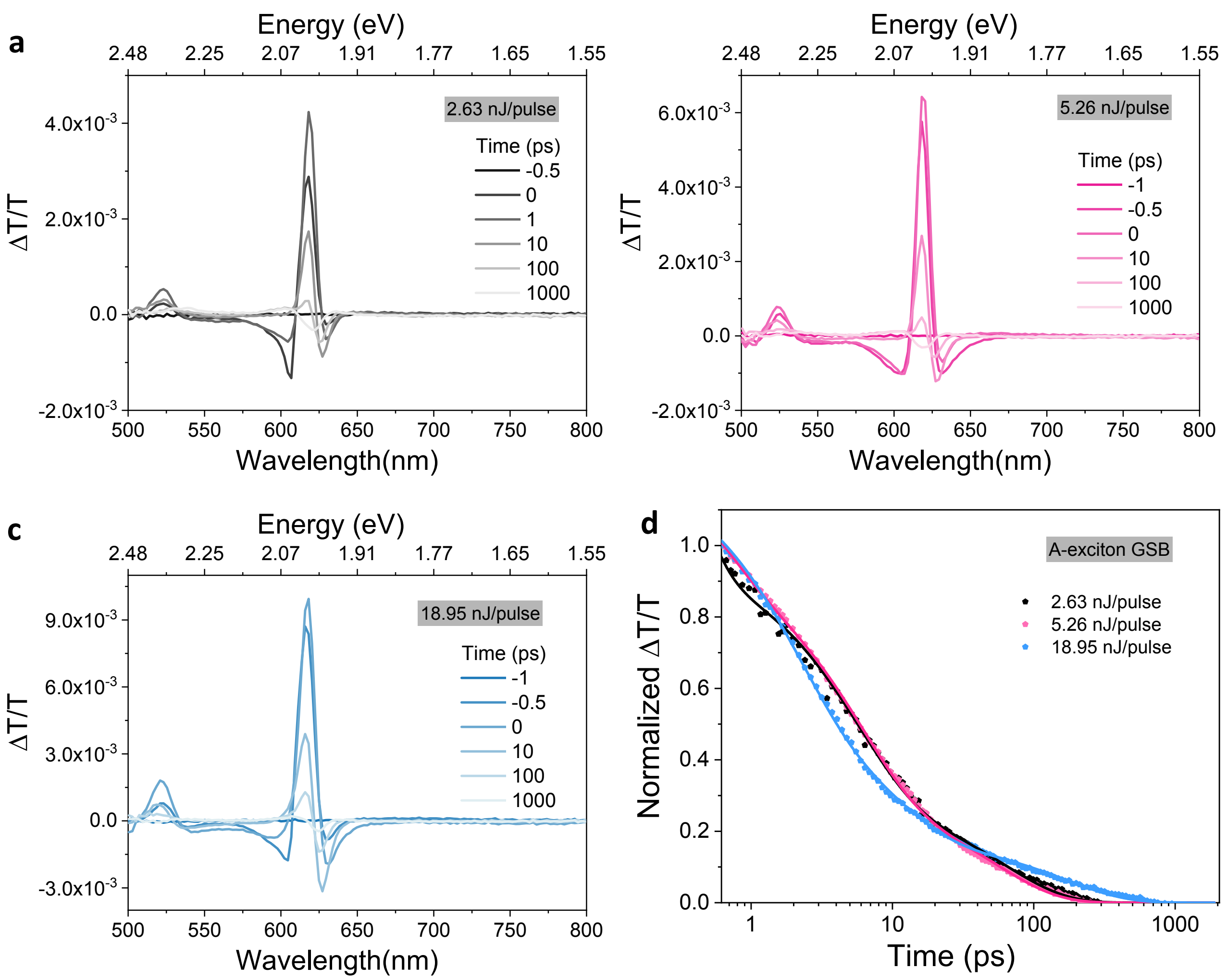

Figure S9. Fluence dependence of A-exciton resonance (excited at 610 nm) in pump-probe measurements. Pump-probe data of mechanically exfoliated untreated monolayer WS_2 sample with **a** 2.63 nJ/pulse, **b** 5.26 nJ/pulse, and **c** 18.95 nJ/pulse. **d** Normalized kinetics of A-exciton GSB of all samples.

Table S6. Fitting results for the rates at 610 nm excitation of mechanically exfoliated untreated WS_2 monolayer sample in pump-probe measurement.

Sample	A_1	τ_1 (ps)	A_2	τ_2 (ps)	A_3	τ_3 (ps)	<τ> (ps)
2.63 nJ/pulse	2.68	0.18	0.67	5.5	0.29	62	6
5.62 nJ/pulse	0.44	0.47	0.68	5.7	0.29	55	14
18.95 nJ/pulse	0.68	1.93	0.37	10.0	0.18	167	29

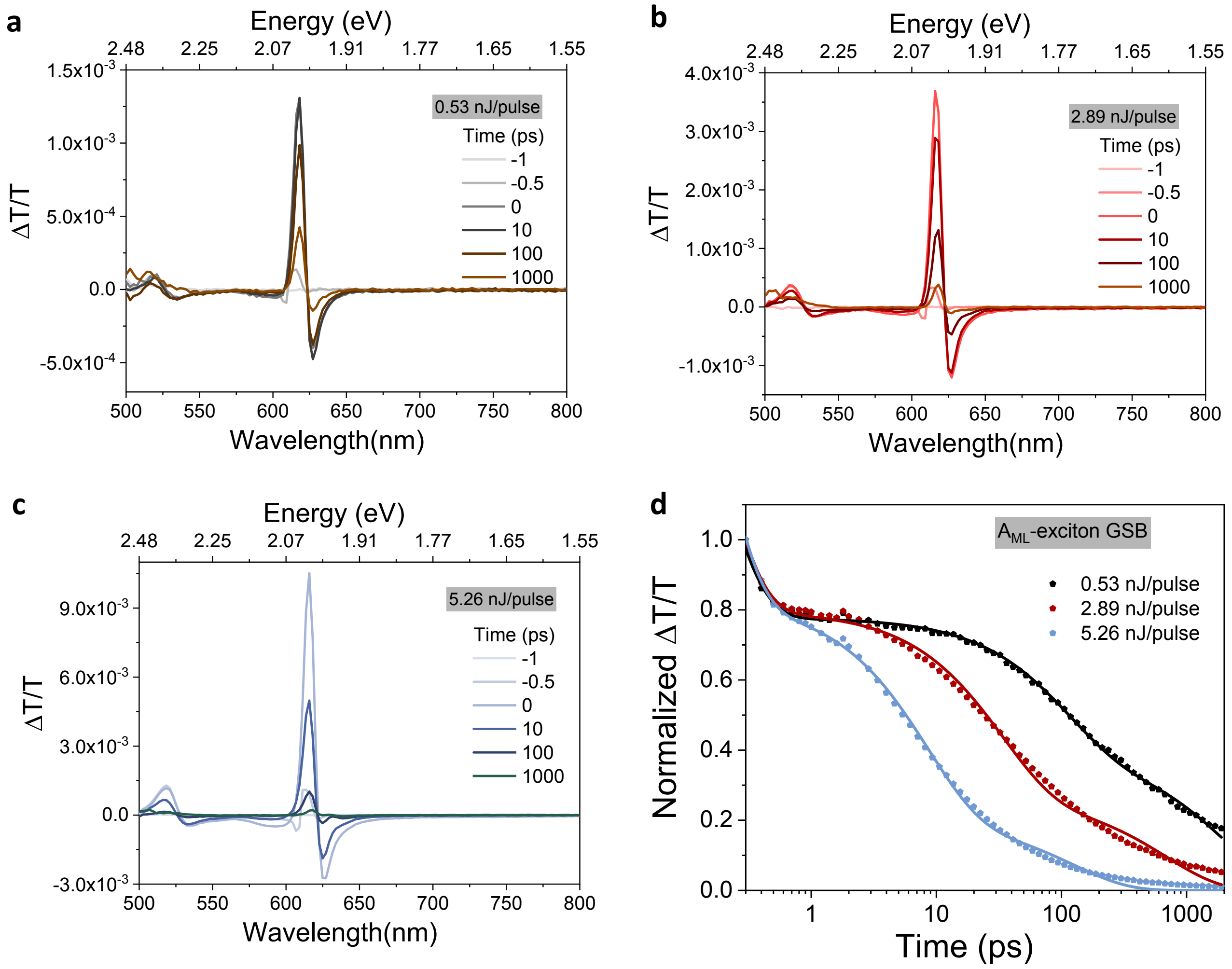

Figure S10. Fluence dependence of A-exciton resonance (excited at 610 nm) in mechanically exfoliated Li-TFSI treated monolayer WS_2 sample. Pump-probe data with **a** 0.53 nJ/pulse and **b** 2.83 nJ/pulse. **c** 5.26 nJ/pulse **d** Normalized kinetics taken at A_{ML}-exciton GSB of all samples.

Table S7. Fitting results for the rates at 610 nm excitation of mechanically exfoliated Li-TFSI treated WS_2 monolayer sample in pump-probe measurement.

Sample	A_1	τ_1 (ps)	A_2	τ_2 (ps)	A_3	τ_3 (ps)	$\langle\tau\rangle$ (ps)
0.53 nJ/pulse	1.77	0.14	0.39	108	0.39	2024	324
2.83 nJ/pulse	2.05	0.13	0.26	688	0.54	32.8	70
5.26 nJ/pulse	2.62	0.12	0.62	8	0.20	126	9

6. Reference

(1) Tainter, G. D.; Hörantner, M. T.; Pazos-Outón, L. M.; Lamboll, R. D.; Āboliņš, H.; Leijtens, T.; Mahesh, S.; Friend, R. H.; Snaith, H. J.; Joyce, H. J., et al. Long-Range Charge Extraction in Back-Contact Perovskite Architectures via Suppressed Recombination. *Joule* **2019**, *3*, 1301–1313. https://doi.org/10.1016/j.joule.2019.03.010.

(2) Allardice, J. R.; Thampi, A.; Dowland, S.; Xiao, J.; Gray, V.; Zhang, Z.; Budden, P.; Petty, A. J.; Davis, N. J. L. K.; Greenham, N. C., et al. Engineering Molecular Ligand Shells on Quantum Dots for Quantitative Harvesting of Triplet Excitons Generated by Singlet Fission. *J. Am. Chem. Soc.* **2019**, *141*, 12907–12915. https://doi.org/10.1021/jacs.9b06584.

(3) Backes, C.; Szydłowska, B. M.; Harvey, A.; Yuan, S.; Vega-Mayoral, V.; Davies, B. R.; Zhao, P. L.; Hanlon, D.; Santos, E. J. G.; Katsnelson, M. I., et al. Production of Highly Monolayer Enriched Dispersions of Liquid-Exfoliated Nanosheets by Liquid Cascade Centrifugation. *ACS Nano* **2016**, *10*, 1589–1601. https://doi.org/10.1021/acsnano.5b07228.
